# Medication Nonadherence and Associated Factors among Heart Failure Patients at University of Gondar Comprehensive Specialized Hospital, Northwest Ethiopia

**DOI:** 10.1155/2023/1824987

**Published:** 2023-01-14

**Authors:** Mohammed Assen Seid, Husien Nurahmed Toleha, Faisel Dula Sema

**Affiliations:** ^1^Department of Clinical Pharmacy, School of Pharmacy, College of Medicine and Health Sciences, University of Gondar, Ethiopia; ^2^Department of Social and Administrative Pharmacy, Wollo University, Ethiopia

## Abstract

**Background:**

Medication nonadherence, being one of the best predictors of hospitalization, increases the mortality rate and hospital readmission and reduces the quality of life of heart failure (HF) patients. Therefore, this study is aimed at assessing medication nonadherence and associated factors among HF patients at the University of Gondar Comprehensive Specialized Hospital, Northwest Ethiopia.

**Methods:**

A cross-sectional study was conducted among 245 adult patients with HF from June to August 2017. The data were collected by using the medication Adherence Report Scale (MARS-5) and then entered and analyzed using SPSS® (IBM Corporation) version 24. Summary statistics were presented using frequency, proportion, and mean. Binary logistic regression analysis was done for identifying factors associated with medication nonadherence with a 95% confidence level and *p* value of less than 0.05.

**Results:**

Among 245 patients with HF, about a quarter (23.7%) of them were medication nonadherent. More than one-third (37%) of HF patients had a history of at least one HF medication discontinuation. Refilling problems (48%) and getting better from the illness (27%) were the most commonly reported reasons for nonadherence. Presence of comorbidity (AOR = 2.761; 95%CI = 1.364, 5.589), taking three or more types of medication (AOR = 2.805; 95%CI = 1.404, 5.60), and being unmarried (AOR = 2.638, 95%CI = 1.279, 5.443) was significantly associated with medication nonadherence.

**Conclusion:**

The self-reported medication nonadherence among HF patients was considerably high. Refilling problems and getting better from the illness were the most commonly reported reasons for nonadherence. The presence of comorbid illness, taking three or more types of medication, and being unmarried was significantly associated factors of medication nonadherence. Awareness creation among patients on the importance of medication adherence and targeted efforts to assess and mitigate reasons for medication nonadherence may be helpful.

## 1. Introduction

Heart failure (HF) is a major and growing public health problem, which affects over 37.7 million people worldwide [1, 2]. In developing regions like sub-Saharan Africa, it is progressively becoming the leading cause of morbidity, mortality, and a great economic burden on patients, payers, and the health care system [3], a significant amount of which is due to a hospital admission related to nonadherence to evidence-based pharmacotherapies [4, 5], which is the most expensive cost element of this ailment [6, 7].

Medication adherence is defined as “the extent to which the patient's actions meet the prescriber's recommendations or expectations” [8]. It is also defined for long-term therapy as “the extent to which a person's behavior – taking medication, following a diet, and/or executing lifestyle changes, corresponds with agreed recommendations from a health care provider” [9–11].

Medication nonadherence is one of the best predictors of hospitalization in HF patients [12]. It contributes a potentially significant burden to the health care system on both personal and public levels [8, 13]. Being among the main predictor of hospital admission, medication nonadherence imposes a noteworthy avoidable cost of HF in the health care system [12, 14]. Medication nonadherence can increase mortality rate, emergency department visits, hospital readmission, and length of hospital stay and reduce the quality of life of HF patients [15–17]. However, only less than 50% of patients are adherent to their medication in long-term therapy for chronic conditions [8–10].

According to the World Health Organization multidimensional adherence model, factors associated with medication adherence may be related to the patient, condition, treatment, healthcare system, and socioeconomic variables [10]. So far, factors associated with medication adherence in HF patients are reported to be age, sex, educational level, ethnicity, social support, outpatient visit, number of comorbidities, functional status, depression, forgetfulness, polypharmacy, and being symptom-free [13, 16, 18]. Unless medication adherence is included as a key component of HF self-care and kept at an optimal level, nonadherence usually limits the benefit of the medicine [8, 17]. Although the majority of factors associated with medication adherence can be subject to intervention, barriers to medication adherence are complex [13, 17, 19]. So, urgent multifactorial and personalized interventions might be needed to improve HF patients' medication adherence [19, 20].

It is obvious that medication nonadherence exists wherever the self-administration of medication is required, regardless of the type of disease, severity of the disease, and accessibility of health care resources. Therefore, accurate adherence assessment is important to implement appropriate medication adherence intervention strategies [8, 9]. Besides, it is important to know the actual extent of the condition in the world [9]. However, due to competing needs in a population severely affected by poverty, much emphasis is not given to addressing the problem of medication adherence in developing countries [9].

In Ethiopia, few published studies have reported the level of medication adherence among heart failure patients either separately [21] or as a part of drug therapy problems [22]. However, due to a variation in the study setting, the level of care, and sociodemographic characteristics such as language differences, the findings might be difficult to extrapolate to this study setting. Therefore, this study is aimed at assessing medication nonadherence and associated factors among HF patients at the University of Gondar Comprehensive Specialized Hospital, Northwest Ethiopia. So, all necessary measures could be taken to confront the problem by all concerned bodies based on the finding of this study.

## 2. Methods and Materials

### 2.1. Study Design

A cross-sectional study was conducted at the University of Gondar Comprehensive Specialized Hospital, from June to August 2017.

### 2.2. Study Setting

The University of Gondar is one of the oldest medical training institutions located in northwest Ethiopia, which is around 750 km far from Addis Ababa (the capital city of Ethiopia). It has one Comprehensive Specialized Hospital, which serves about 7 million urban and rural populations around the North Gondar administrative zone. The hospital has more than 500 beds for inpatient services for the internal medicine, pediatrics, surgery, and gynecology and obstetrics wards. Also, each department has outpatient departments (OPD) in which outpatient care is delivered for patients with new and chronic illnesses like HF. HF is the second most common cardiovascular illness in the institution [23]. Outpatient service for HF patients who have chronic follow-up is mostly given one out of 5 working days depending on their appointment schedule; however, patients can get nonscheduled service on any day of the five working days. The health care team consists of senior physicians, residents, interns, nurses, and pharmacists.

### 2.3. Population

The source population of this study was all patients who were on medication therapy for HF at the University of Gondar Comprehensive Specialized Hospital chronic OPD, whereas the study population was all patients who were on medication therapy for HF at the University of Gondar Comprehensive Specialized Hospital chronic OPD from June to August 2017.

### 2.4. Inclusion and Exclusion Criteria

All adult patients aged greater than or equal to 18 years who were stable with a medication therapy of HF and had a scheduled follow-up at the chronic OPD within the study period regardless of the type and class of HF were included in this study. Patients had to meet the Framingham criteria for the diagnosis of heart failure (presence of either two major criteria or a combination of one major criterion and two minor criteria). Patients who were critically ill and unable to communicate during the data collection time were excluded.

### 2.5. Sample Size and Sampling Procedure

During the study, 1100 HF patients had a scheduled follow-up at chronic OPD. In most cases, patients were appointed every one, two, or three months based on their clinical condition and residence (rural or urban). From a total of 301 patients selected by using a simple random sampling technique, 245 patients fulfilled the inclusion criteria and gave consent to participate.

### 2.6. Data Collection Method and Procedures

The data were collected by three nurses through a face-to-face interview using a structured questionnaire under the supervision of the principal investigator. Patients were interviewed during their scheduled follow-up at chronic OPD. For patients who did not come in the first scheduled follow-up, the next scheduled follow–up was considered. Patient's sociodemographic variables like age, sex, marital status, place of residence, educational level, reasons for drug discontinuation, and medication adherence were collected from the patient interview. Patient charts were also reviewed for obtaining supplemental clinical data like chronic comorbidities, types and duration of HF medication use, type and class of heart failure, and history of hospitalization. A validated tool, the Medication Adherence Report Scale (MARS-5) [24, 25], was used for assessing medication adherence. The MARS-5 is assumed to be informative for identifying reasons for nonadherence in routine clinical practice. It comprises 5 items for self-reporting common patterns of nonadherent behavior. Each item was rated on a 5-point Likert scale (with 1 = always, 2 = often, 3 = sometimes, 4 = rarely, and 5 = never) [24, 25]. The range of the MARS-5 total score is between 5 and 25. A higher score on the MARS-5 represents better medication adherence [24, 25]. Many previous self-report adherence scales have recommended separating the population into two groups (adherent and nonadherent) [26]. Cut-off points ranging from 20 to 25 have been reported for the MARS-5 [24, 27], so that we dichotomized the MARS-5 sum score into either ≥20 or<20 from a total score of 25 as medication adherent and nonadherent, respectively. Even though the questionnaire was prepared based on a previously validated tool, it was sent to two facility members and a physician to assess the face validity because of the presence of slight modification. The questionnaire had been pretested by 5% (23 HF patients) of the study samples before data collection was started. For internal consistency, the alpha Cronbach test which had an index of 0.85 was used. The questionnaire was translated into Amharic language and back-translated to the English language to minimize possible translation errors by experts in the area. Data were not recollected from the individuals who participated to pretest the questionnaire. The data collected for pretesting the questionnaire was not included in the final analysis too.

### 2.7. Data Quality Control

For assuring the quality of the data, all data collectors had been trained before the data collection was started on the objective of the study, the contents of the questionnaire, and ethical issues. Data collection was supervised throughout the data collection period. The questionnaire was pretested on 5% of the sample size.

### 2.8. Data Processing and Analysis

All the collected data were checked for completeness and consistency of responses manually. After cleaning, data were coded and entered into EpiData version 3.1 and analyzed by using the Statistical Package for Social Sciences (SPSS®) IBM Corporation version 24. The descriptive statistics were presented by using frequency, proportion, mean, and median. The binary logistic regression analysis was done to determine the presence of statistically significant associations between the dependent variable and the independent variables. The model was tested by using Hosmer–Lemeshow goodness of fit (chi − square = 11.983; df = 8; sig = 0.152). After checking for multicollinearity and outliers, all independent variables with *p* < 0.2 in the bivariate binary logistic regression analysis were entered for multivariate binary logistic regression analysis using the enter method. The crude odds ratios (COR) and adjusted odds ratios (AOR) were used to determine the strength and direction of association between the dependent and independent variables in bivariate and multivariate regression, respectively. Independent variables with a *p* value of less than 0.05 were considered as significantly associated factors.

### 2.9. Ethics Approval

Ethical clearance approval was obtained from the Ethics Committee of University of Gondar, School of Pharmacy, Department of Clinical Pharmacy with a reference number SoPs/908/2017. This study has been done by following all methods in accordance with the relevant guidelines and regulations. After participants were informed about the objective of the study, both written and verbal consent had been obtained from each study participant before data collection was started. During the consent process, we provided information for the study participants regarding the purpose of the study, why and how they were selected to involve in the study, and they had the right to not participate and could withdraw from the study at any time if they were not interested. They were all assured that their care would not be affected in any way due to refusing consent to participate in the study. Participants were also assured of the confidentiality of the information obtained in the course of this study by not using personal identifiers.

## 3. Results

Among 245 HF patients, the majority (57%) of the study participants were above the age of 45. More than two-thirds (67%) of the study participants were female, and the majority (60%) of HF patients had no formal education in this study. Hypertension was identified as the major (21.6%) comorbid condition of HF patients. The majority (65%) of HF patients had a hospitalization history, and 46% of the study participants were using three and more types of HF medication. Around half (47.8%) of patients had New York Heart Association (NYHA) functional class IV HF. Nearly two-thirds (63.3) of patients had HF with ejection fraction (EF) < 40 (Table 1).

### 3.1. HF Patients' Level of Medication Adherence

**Figure 1:**
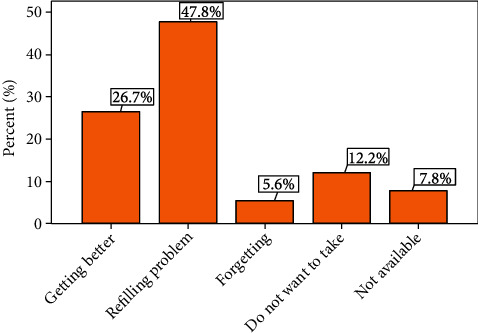
Reasons reported by HF patients to discontinue their medication.

The sum of the mean and median scores of MARS-5 was 21.23 (standard deviation (SD) = 2.54) and 22 (range = 12 − 25), respectively. The highest individual mean score (mean = 4.86; SD = 0.35) was obtained for the statement “I change the dosage of my HF medication”, whereas the lowest mean score (mean 3.38; SD = 0.90) was obtained for the statement “I stop taking my HF medication for a while”, the overall mean score being 21.23 (mean = 2.54; SD = 12 − 25). Out of 245 HF patients, around a quarter (23.67%) of HF patients were nonadherent to their medication (Table 2). Around 37% (*N* = 90) of HF patients had a history of stopping at least one of their HF medications. Of these, the most common reasons of nonadherence reported by the patients were the inability of refiling upon finishing the medicine (48%) and getting better from the illness (27%) (Figure 1).

As presented in Table 3, the binary logistic regression analysis showed that HF patients with chronic comorbid diseases were around 3 times (AOR = 2.761; 95%CI = 1.364, 5.589; *p* value =0.005) more likely to be nonadherent to their medication than patients with no chronic comorbid diseases. HF patients who were taking three and more than three types of medication were about 3 (AOR = 2.805; 95%CI = 1.404, 5.60; *p* value =0.003) times more likely to be nonadherent to their medication than their counterparts. Similarly, unmarried HF patients were more than 2 times (AOR = 2.638, 95%CI = 1.279, 5.443; *p* value =0.009) more likely to be nonadherent to their medication than married patients (Table 3).

AOR: adjusted odds ratio; COR: crudes odds ratio; CI: confidence interval; HF: heart failure.

## 4. Discussion

Medication adherence is a pivotal point in the medication management of HF patients. However, a significant number of HF patients may have difficulty adhering to their HF medications [13, 16, 28–30]. So, this study was aimed at assessing medication nonadherence and associated factors among HF patients at the University of Gondar Comprehensive Specialized Hospital, northwest Ethiopia.

The result of this study implicates that around a quarter (23.67%) of HF patients were nonadherent to their medication. It is consistent with previous studies done by Lee et al (22.1%). However, it is relatively lower than the study done by Fayaz et al. (72.7%) and Silva et al. (72.7%). Similar studies done by Amininasab et al. and Aggarwal et al. reported that the majority of patients with HF had low medication adherence [13, 16]. The difference may be to the variation in the sociocultural contexts, adherence measurement tools, and the cutoff point used to classify the adherence level. Generally, medication nonadherence among HF patients is high. It, though modifiable, may result in an increased mortality rate, emergency department visits, hospital readmission, worse cardiac event-free survival, and reduction of quality of life among HF patients [15–17, 29–33]. So, deliberate efforts to assess and improve adherence should be incorporated and become an integral part of daily patient management [31].

In this study, the reasons reported by HF patients to discontinue their medication were refilling problems, a sense of getting better, do not want to take medication, nonavailability, and forgetting to take the medication. Similarly, in the studies done by Aggarwal et al. [13], Mujtaba et al. [28], and Shah et al. [34], forgetfulness, being symptom-free, nonavailability of medication, and patients do not want to take medication were among the reasons for discontinuation of HF medications [13, 28, 34]. Encouraging patients for timely refilling and an arrangement of appointments in consideration of patients' convenience and preference may decrease nonadherence due to refiling problems. Also, awareness creation among patients on the need for continuing medication despite the resolution of symptoms and a sense of getting better in case of chronic illness is necessary. Convincing patients of the importance of taking medication for the treatment of HF through a patient-centered approach may also be helpful. The use of new techniques like an alarm on their cell phone and the participation of their family may help to remind patients to take their medication. The commonly used medicine for the treatment of HF should also be kept available, always.

HF patients with chronic comorbid diseases were around 3 times more likely to be nonadherent to their medication than patients with no chronic comorbid diseases. Previous studies also reported that HF patients who had chronic disease comorbidity showed nonadherence to their medication [16, 35, 36]. Contrarily, Bagchi et al. reported that comorbidity was associated with higher medication adherence [37]. The difference may be due to the variation in the types of comorbidity in different studies. For example, previous studies showed that the presence of diabetes mellitus as a comorbidity increases adherence; however, the presence of hypertension depression increases nonadherence to medication [38]. So, HF patients with comorbidities should be given special attention concerning their medication adherence.

Similarly, HF patients who were taking three and more types of medication were about 3 times more likely to be nonadherent to their medication than their counterparts. Likewise, the majority of previous studies reported that when the types of medication increased, patients' adherence levels decreased [16, 35, 39, 40]. This may be because patients who are taking multiple types of medications will face difficulty to manage their multiple types of medications, increasing medication side effects and thereby decreasing their adherence level [41]. Therefore, decreasing the number of medications through different techniques like using a fixed-dose combination, when possible, may increase patients' medication adherence.

Similarly, HF patients who were unmarried were more than 2 times more likely to be nonadherent to their medication than married patients. This finding is consistent with previous studies done by [33, 42]. It was also demonstrated that patients who lived with their families had higher adherence scores in a previous study [38]. This implies that the incorporation of social support as a possible strategy for improving medication adherence of HF patients may have a considerable contribution.

### 4.1. Study Limitations

This study has some limitations that should be considered while interpreting the findings. The relatively small sample size, being a single-center study, measurement of adherence by the means of a self-report questionnaire, presence of social desirability, and recall bias may affect the generalizability of the study to the general population of HF patients. Moreover, this research has not tested some lifestyle factors like alcohol use, smoking, body mass index, individual drugs and comorbidities, and blood pressure of the participants, which would have better explained the study findings.

## 5. Conclusions

The self-reported medication nonadherence among HF patients was considerably high. Refilling problems, a sense of getting better from the illness, not wanting to take medication, nonavailability, and forgetting to take the medication were the reasons for medication discontinuation. The presence of comorbid illness, taking three or higher types of medication, and being unmarried was significantly associated factors of medication non-adherence.

Therefore, all necessary efforts should be made to improve the medication adherence of HF patients. Activities to decrease medication nonadherence should be targeted at mitigating refiling problems, awareness creation of patients regarding the necessity of taking and continuing medication despite symptom resolution and getting better from the illness, reminding patients to take their medication, and availing of the commonly used medication for the treatment of HF. Special attention should also be given to patients having chronic disease comorbidities, taking three or more types of medication, and being unmarried.

## Figures and Tables

**Table 1:** Sociodemographic and clinical characteristics of HF patients at the University of Gondar Hospital.

Variables	Categories	*N* (%)
Age^∗^ (in years)	<45	105 (42.9)
	≥45	140 (57.1)

Sex	Female	163 (66.5)
	Male	82 (33.5)

Marital status	Married	125 (51.0)
	Single	50 (20.4)
	Divorce	28 (11.4)
	Widowed	42 (17.1)

Educational level	No formal education	149 (60.8)
	Primary school [1–8]	52 (21.2)
	Secondary school [9–12]	26 (10.6)
	College/university	18 (7.3)

Place of residence	Rural	114 (46.5)
	Urban	131 (53.5)

Presence of chronic comorbidity	No	136 (55.5)
	Yes	109 (44.5)

Type of comorbidity	HTN	53 (21.6)
	CKD	18 (7.3)
	DM	4 (1.6)
	HTN + CKD	21 (8.6)
	Hyperthyroidism	13 (5.3)

Duration since started to take HF medications	<5 years	158 (64.5)
	5-10 years	65 (26.5)
	>10 years	22 (9.0)

Hospitalization history from the 1^st^ diagnosis	No	87 (35.5)
	Yes	158 (64.5)
Ejection fraction (EF)	EF < 40	155 (63.3)
	EF ≥ 40	90 (36.7)
New York Heart Association (NYHA) functional class	Class I	18 (7.3)
	Class II	33 (13.5)
	Class III	77 (31.4)
	Class IV	117 (47.8)

Types of medication the patients were taking	ACEIs	4 (1.6)
	Diuretics	62 (25.3)
	B-blockers	3 (1.2)
	ACEIs + diuretics	42 (17.1)
	ACEIs + B-blockers	4 (1.6)
	Diuretics + B-blockers	17 (6.9)
	Three or more than three types of drugs	113 (46.1)

Total number of prescribed medication (per day/patient) (mean = 3.1; SD = 1.1; range 1-6)		

^∗^The mean age = 47.87 ≈ 45 with SD = 19.64 and range 18-89; SD: standard deviation; HTN: hypertension; CKD: chronic kidney disease; DM: diabetes mellitus; ACEIs: angiotensin-converting enzyme inhibitors; *N*: frequency; %: percentage.

**Table 2:** HF patients' level of adherence to their medication at the University of Gondar Comprehensive Specialized Hospital, northwest Ethiopia.

MARS-5^∗^ items	Mean score (SD)	Median (range)
I change the dosage of my HF medication	4.86 (0.35)	5 (4-5)
I forget to take my HF medication	4.76 (0.43)	5 (4-5)
I use my HF medication less than is prescribed	4.74 (0.72)	5 (1-5)
I decide to skip one of my HF medication dosages	3.50 (1.68)	4 (1-5)
I stop taking my HF medication for a while	3.38 (0.90)	4 (1-5)
The overall score	21.23 (2.54)	22 (12-25)
Medication adherence	Frequency (%)	95% CI
Medication adherent	187 (76.3%)	70.7, 81.2
Medication nonadherent	58 (23.7)	18.8, 29.3

^∗^Medication Adherence Report Scale (MARS-5); %: percent; CI: confidence interval; SD: standard deviation.

**Table 3:** Binary logistic regression analysis of factors associated with medication nonadherence among HF patients at University of Gondar Comprehensive Specialized Hospital, northwest Ethiopia.

Variables	HF medication adherence		COR (95% CI)	AOR (95% CI)	*p* value
	Nonadherent	Adherent			
*Age (in years)*					
<45	22	83	0.766 (0.342, 0.910)	0.929 (0.425, 2.031)	0.884
≥45	36	104	1	1	
*Sex*					
Female	38	125	0.942 (0.506, 1.754)	1.030 (0.493, 2.149)	0.937
Male	20	62	1	1	
*Place of residence*					
Rural	28	86	1.096 (0.608, 1.977)	1.526 (0.738, 3.157)	0.254
Urban	30	101	1	1	
*Marital status*					
Unmarried	35	85	1.826 (1.002, 3.326)	2.638 (1.279, 5.443)	0.009
Married	23	102	1	1	
*Educational level*					0.183
No formal education	35	114	1.623 (0.665, 3.961)	1.824 (0.602, 5.522)	0.288
Primary school [1–8]	16	36	2.349 (0.864, 6.384)	2,786 (0.924, 8.394)	0.69
High school and above	7	37	1	1	
*Chronic disease comorbidity*					
Yes	37	72	2.814 (1.527, 5.185)	2.761 (1.364, 5.589)	0.005
No	21	115	1	1	
*Duration since started to take HF medications*					0.930
<5 years	37	121	1	1	
5-10	15	50	0.800 (0.266, 2.407)	1.105 (0.323, 3.775)	0.874
>10 years	6	16	1.226 (0.448, 3.360)	1.051 (0.347, 3.185)	0.930
*Number of medications the patients were taking*					
Three or more types of drugs	39	74	3.134 (1.683,5.837)	2.805 (1.404, 5.604)	0.003
Less than three types of drugs	19	113	1	1	

## Data Availability

All data generated or analyzed during this study are included in this manuscript.
